# Sequencing and *De Novo* Assembly of the Complete Chloroplast Genome of the Peruvian Carrot (*Arracacia xanthorrhiza* Bancroft)

**DOI:** 10.1128/genomeA.01519-16

**Published:** 2017-02-16

**Authors:** Javier Santiago Alvarado, Diane Hinojosa López, Isaury Maldonado Torres, María Margarita Meléndez, Rosalinda Aybar Batista, Vivek K. Raxwal, Juan A. Negrón Berríos, Alok Arun

**Affiliations:** a Institute of Sustainable Biotechnology, Department of Science and Technology, Inter American University of Puerto Rico, Barranquitas, Puerto Rico; b Plant Molecular Biology Group, Central European Institute of Technology, Brno, Czech Republic

## Abstract

*Arracacia xanthorrhiza* is an important secondary food crop in South America and Puerto Rico. The lack of crop protection and improvement strategies leads to infections damaging the storage roots. Here, we report the annotated complete chloroplast genome sequence of *A. xanthorrhiza* as a step toward developing genomic resources for this crop.

## GENOME ANNOUNCEMENT

The Peruvian carrot (*Arracacia xanthorrhiza* Bancroft), commonly known as apio, is a tuberous perennial crop in the family *Apiaceae* originating from the Andes (1–3). *A. xanthorrhiza* is a secondary food crop for over 100 million people, mainly in South America and Puerto Rico. Starch-rich storage roots are the main food product, the rootstock and leaves are used as animal feed, and the aerial stems are used as propagules (1, 4). The crop has low-input requirements and can be grown in a variety of frost-free tropical highland environments (2). However, in the absence of preventive management practices and proper handling, infections caused by pathogens severely damage the storage roots (5). The lack of certainty about the agents and the process of infection pose a major obstacle to understanding and preventing infection at its very onset. Despite the economic and agricultural importance of *A. xanthorrhiza*, any biotechnological strategy aimed at improving the yield or protecting the crop from pest damage is nonexistent, partly due to the lack of genomic resources. Here, we present the sequence and annotated chloroplast genomic DNA (cpDNA) of *A. xanthorrhiza*. Chloroplast genomes have been used extensively for DNA barcoding (6, 7), phylogenetic analyses (8), plastid transformation (9), and gene silencing studies (10). The availability of the chloroplast genome sequence of *A. xanthorrhiza* would be the first major step toward its bioengineering.

In the present study, cpDNA was extracted from leaf tissues of *A. xanthorrhiza* according to the protocol described in Shi et al. (11) with minor modifications. Approximately, 1 ng of cpDNA was used to prepare sequencing libraries using Illumina’s NexteraXT DNA sample prep kit. Paired-end (2 × 150 bp) sequencing reactions were performed on Illumina’s MiSeq platform at the Institute of Sustainable Biotechnology, IAUPR, Puerto Rico. The pair-end reads were trimmed for adapter and low-quality reads (Phred score <30) using Trim Galore (http://www.bioinformatics.babraham.ac.uk/projects/trim_galore/). The resulting 7.9 million sequencing reads generated an estimated 1,101-fold chloroplast genome coverage. The *de novo* assembly of the filtered pair-end reads was performed using NOVOPlasty (12). Two annotation tools, CPGAVAS (13) and DOGMA (14), were used to annotate the cp genome. Finally, protein-coding genes were manually annotated.

The current investigation suggests that the *A. xanthorrhiza* circular chloroplast genome is 143,989 base pairs (bp) in length with 37.48% G+C content. The cp genome is divided into (a) large single copy (LSC) (49,169 bp), (b) small single copy (SSC) (17,439 bp), and (c) two inverted regions (31,370 bp). The cp genome encoded 106 manually curated unique genes consisting of 71 protein-coding genes, seven rRNA genes, and 28 tRNA genes. Exons of five genes, namely, *ycf3*, *clpP*, *rpsl2*, *ndhA*, and *ndhB*, were interrupted with intron sequences. The genome architecture of the *A. xanthorrhiza* cp genome shows strong similarity to cp genome of closely related *Apiaceae* crop carrot, *Daucus carota* (15). The availability of the cp genome sequence of *A. xanthorrhiza* will be an asset for studying the genetic diversity of various apio landraces and their closely related wild relatives, and will contribute to a better understanding of genomics of this economically important crop.

### Accession number(s).

The chloroplast DNA genome sequence has been deposited in GenBank under accession number KY117235.
